# Data collected in an integrated ecological survey of rotifer communities and corresponding environmental variables in the highly polluted Haihe River Basin, China

**DOI:** 10.1016/j.dib.2017.12.062

**Published:** 2018-01-03

**Authors:** Wei Xiong, Jie Li, Yuzhan Yang, Weimin Wang, Baoqing Shan, Aibin Zhan

**Affiliations:** a Research Center for Eco-Environmental Sciences, Chinese Academy of Sciences, 18 Shuangqing Road, Haidian District, Beijing 100085, China; b University of Chinese Academy of Sciences, Chinese Academy of Sciences, 19A Yuquan Road, Shijingshan District, Beijing 100049, China; c College of Fisheries, Huazhong Agricultural University, 1 Shizishan Street, Wuhan 430070, China

**Keywords:** Environmental pollution, Nitrogen, Phosphorus, Rotifer, Stream

## Abstract

Here we presented two datasets (biological and environmental datasets) collected in a comprehensive large geographical scale (approximately 1.1×10^5^ km^2^) survey of rivers/streams in the Haihe River Basin (HRB), which has become the most polluted river basin in past two decades in China. The survey selected a total of 94 representative sampling sites in the plain region of HRB, where environmental pollution is more severe than the mountain region. The biological dataset contains the information on the identified rotifer species and their abundance, while the environmental dataset provides the measured environmental variables at each sampling site. Based on this ecological survey, we identified a total of 91 rotifer species and their abundance, as well as abundance of two crucial taxonomic groups on rotifers’ food webs (i.e., protozoans and crustaceans), and also presented seven environmental variables, particularly those associated with nitrogen and phosphorus pollution.

**Specifications Table** [*Please fill in right-hand column of the table below.*]

**Table:** 

Subject area	*Ecology*
More specific subject area	*Ecology of Environmental Pollution*
Type of data	*Table, Figure*
How data was acquired	*Microscope, Chemical analysis*
Data format	*Raw data, analyzed data*
Experimental factors	–
Experimental features	*Rotifer samples were preserved in 5% formaldehyde for microscopic examination. Water samples were added 1% sulfuric acid in the field for measurement of TN and TP; Water samples were filtered by 0.45μm glass microfiber filters for measurement of NO*_*3*_*-N, NH*_*4*_*-N and SRP.*
Data source location	*The plain region of the Haihe River Basin, covering cities of Beijing and Tianjin, as well as Hebei, Henan and Shandong provinces in the North China.*
Data accessibility	*Data is presented in this article.*
Related research article	*Data is related to articles published and in review*[1], [2]

**Value of the Data**

1. Both datasets from a large geographical scale survey (approximately 1.1×10^5^ km^2^) provide baselines for the river/stream status, as well as references for meta-surveys on both biological and environmental factors in future studies in the Haihe River Basin.
2. As many rotifer species have been reported as sound environmental indicators of aquatic ecosystem health, the species identified here, as well as associated environmental factors, can provide promising indicators for environmental pollution in Northern China.
3. The datasets improve the overview of the rotifer biodiversity and geographical distributions, as well as geographical distributions of environmental pollutants in the Haihe River Basin, China.
4. Rotifers, which are an important taxonomic group of freshwater biodiversity, may help understand how river/stream biodiversity is influenced by different types and/or degrees of environment pollution.
5. Both datasets of environmental variables and rotifer biodiversity are useful for water quality assessment and potential influence of environmental pollution on biodiversity in many environmental management programs.

## 1 Data

The river ecosystem is likely the most impacted one on the Earth, as rivers/streams are heavily influenced by intensive anthropogenic activities such as pollutant release [3]. Increasing anthropogenic activities have become a major threat to freshwater biodiversity [4]. In China, the Haihe River Basin (HRB) has become the most polluted water ecosystem in the past two decades based on the Report on the State of the Environment in China, 1997-2016. Given an increasing level of environmental pollution in river ecosystems in both China and globally, the study of causes and consequences for biodiversity loss in stressed ecosystems is the prerequisite for management and restoration programs.

Rotifers are one of the dominant microscopic animal groups in river ecosystems [1], [5]. They have been recognized as ecological indicators for environmental changes such as environmental pollution [1], [5]. Investigating biodiversity patterns and geographical distributions of rotifers in stressed river ecosystems would be conducive to understanding biological responses to water pollution and ecological mechanisms for structuring rotifer communities. However, the large geographical scale survey was hindered by laborious field and laboratory works, as well as the lack of reference in both biological and environmental data [5], [6], [7], [8]. Consequently, most studies just focused on single streams, lakes or ponds, or on some specific rotifer species [5], [6], [7], [8].

Based on a large geographical survey (approximately 1.1×10^5^ km^2^) in the plain region of HRB, here we presented the datasets containing the information of both rotifer species and environmental variables at 94 sampling sites. We aim to provide the basic datasets in polluted freshwater ecosystems for testing hypotheses in ecological and environmental studies, and facilitating decision making, environmental restoration and biodiversity recovery in management programs.

We identified a total of 91 rotifer species across 94 sampling sites in the plain region of HRB. These species belonged to ten families, including Asplanchnidae, Brachionida, Dicranophoridae, Gastropodidae, Lecanidae, Notommatidae, Philodinidae, Synchaetidae, Testudinellidae, Trichocercidae. The data of these species and their abundance in each sample were exclusively available in this open access data. In addition, we presented the corresponding environmental variables, including water temperature (T), secchi disk depth (SD), total nitrogen (TN), total phosphorus (TP), nitrate nitrogen (NO_3_-N), ammonia nitrogen (NH_4_-N), soluble reactive phosphorus (SRP), as well as abundance of protozoans and crustaceans at the 94 sampling sites in an open access way.

## 2 Experimental design, materials, and methods

### 2.1 Site selection

The Haihe River basin, covering 310,000 km^2^ and consisting of more than 300 tributaries, is one of the largest and most polluted water systems in the North China. Compared to the mountain region of HRB, the plain region suffers more serious environmental pollution. The plain region covers approximately 110,000 km^2^ and supports both a large area of farmlands and the fastest growing economic regions, such as Beijing, Tianjin. The non-point and point chemical pollutions have largely degraded the ecological integrity, and most rivers/streams in HRB have become highly eutrophic over the past two decades.

**Fig. 1:**
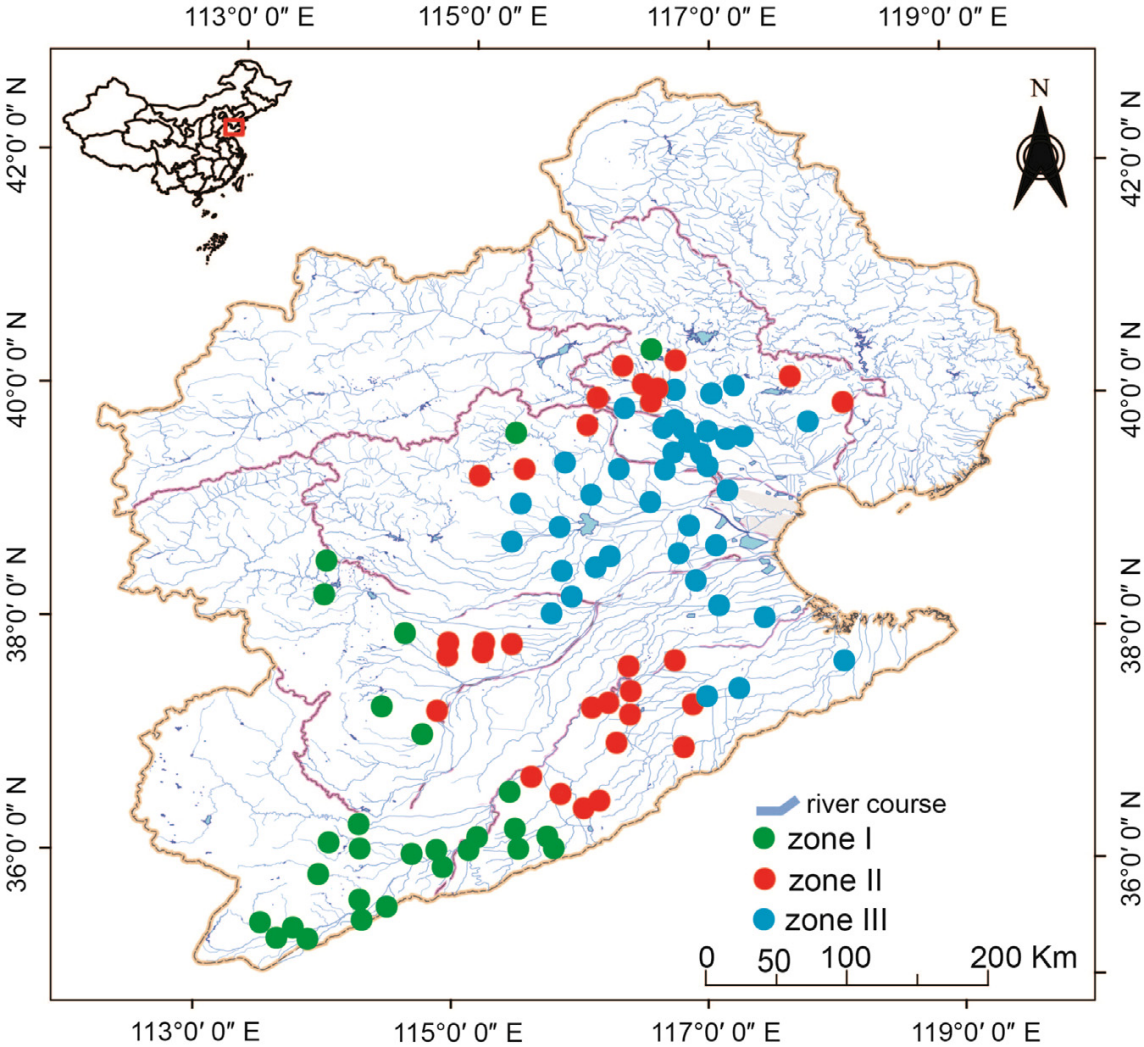
Sampling sites in the plain region of the Haihe River Basin [2].

To conduct a comprehensive survey at a large geographical scale, the plain region was characterized based on the geographical and hydrological features of all tributaries using ArcGIS version 10.0 (ESRI Company, USA). Briefly, according to the upper, middle and lower reaches of each river, the plain region was generally divided into three zones, i.e., zones I–III (Fig. 1). In addition, the zones I-III are located in the altitude of 40–369, 21–39, and 6–20 m above sea level, respectively. To choose the representative sampling sites, the gradient of chemical pollution across the plain region of HRB was taken into consideration and thus a total of 94 sampling sites were selected out of 421 analyzed locations, including 28, 28 and 38 sites in zones I–III, respectively (Fig. 1). The selected 94 sampling sites covered the entire plain region of HRB.

### 2.2 Sample collection and analysis

In each sampling site, 30 L water sample was collected and filtered through a 20 μm mesh net. All collected samples were preserved in 5% formaldehyde (final concentration) with a final volume of 100 mL. Meanwhile, to obtain environmental variables at each sampling site, water temperature (T) and Secchi disk depth (SD) were determined in situ. Two parallel 500 mL water samples were collected, and one of which was filtered through a 0.45 μm glass microfiber filter, preparing for the measurement of soluble reactive phosphorus (SRP), nitrate nitrogen (NO_3_-N), and ammonia nitrogen (NH_4_-N) in the laboratory. Another water sample was added 1% H_2_SO_4_ to keep pH < 2, preparing for total nitrogen (TN) and total phosphorus (TP) analyses in the laboratory (Table 1, Table S1). These five variables were measured by the Ultraviolet-visible Spectroscopy (Shimadzu, Japan). In addition, longitude, latitude, and altitude of each site were recorded in the field by a Global Positioning System (Table 2).

**Table 1:** The mean and range of environmental variables measured in each of the three zones in the Haihe River Basin [2].

	zone I		zone II		zone III	
	mean	range	mean	range	mean	range
T (°C)	24.7	17.0-28.0	25.2	11.5-30.0	24.7	21.0-31.0
SD (cm)	209	12-3000	37	15-85	64	15-500
TP (mg/L)	0.775	0.012-4.120	1.926	0.001-6.378	1.53	0.048-4.828
SRP (mg/L)	0.532	0-2.414	1.3	0-5.158	1.038	0-3.243
TN (mg/L)	8.142	0.586-27.016	12.721	1.787-29.994	11.206	1.792-24.990
NO_3_-N (mg/L)	0.939	0.067-6.040	1.614	0-6.330	1.319	0.340-4.428
NH_4_-N (mg/L)	4.605	0.356-9.532	3.719	0.066-16.545	4.632	0.085-19.525

**Table 2:** The records of longitude, latitude, and altitude of each sampling site.

Sample_ID	Longitude	Latitude	Altitude (m)
I_101	116.16	39.89	80
I_103	116.62	40.30	44
I_128	114.13	36.09	126
I_129	114.15	36.10	145
I_132	114.20	36.29	140
I_136	114.19	37.01	244
I_138	114.13	38.04	214
I_144	114.01	38.32	158
I_40	114.95	36.78	43
I_43	114.43	36.11	72
I_44	114.64	36.00	57
I_45	114.61	35.85	57
I_46	113.44	35.26	82
I_47	113.48	35.28	80
I_48	113.77	35.32	72
I_49	113.75	35.35	73
I_50	114.27	35.49	66
I_51	114.28	35.51	66
I_52	114.28	35.51	66
I_54	113.75	35.93	369
I_55	115.48	36.12	45
I_56	115.47	36.14	42
I_57	115.25	36.10	48
I_58	115.16	36.08	47
I_59	115.06	36.11	45
I_60	115.04	36.11	47
I_61	115.32	36.54	50
I_90	114.77	37.71	40
II_104	116.66	40.28	39
II_105	116.53	40.06	21
II_106	116.49	40.08	30
II_107	116.46	40.13	35
II_108	116.41	40.15	34
II_120	116.96	39.78	21
II_125	116.75	39.82	21
II_126	116.70	39.90	21
II_127	116.65	39.93	29
II_161	116.14	39.47	31
II_162	116.14	39.45	30
II_187	117.72	40.02	24
II_63	115.96	36.41	39
II_64	115.98	36.42	34
II_65	116.04	36.73	33
II_67	115.68	36.82	37
II_68	115.69	36.85	33
II_69	116.32	36.81	30
II_70	116.32	36.81	27
II_74	116.24	37.36	28
II_75	116.23	37.36	27
II_78	116.41	37.34	26
II_82	115.71	37.68	23
II_84	115.49	37.62	25
II_85	115.43	37.60	27
II_86	115.05	37.52	28
II_88	115.09	37.49	26
II_9	116.24	37.37	29
III_100	115.45	38.70	18
III_11	117.37	37.85	12
III_110	117.92	39.80	13
III_111	117.90	39.77	8
III_112	117.21	39.57	6
III_113	117.18	39.59	11
III_114	116.97	39.52	9
III_115	116.95	39.54	11
III_116	116.94	39.72	16
III_117	116.93	39.75	16
III_118	116.92	39.73	19
III_119	116.99	39.78	17
III_121	116.94	39.77	15
III_122	116.93	39.80	18
III_123	116.92	39.83	19
III_124	116.77	39.80	20
III_15	117.88	37.54	7
III_16	116.88	38.70	9
III_23	117.11	39.44	9
III_29	117.52	39.36	6
III_3	116.87	38.50	11
III_31	117.40	39.77	9
III_32	117.38	39.78	8
III_4	116.83	38.49	9
III_7	116.77	38.19	12
III_71	116.75	37.00	20
III_72	116.79	37.00	19
III_73	115.98	38.69	11
III_79	116.84	37.49	17
III_80	116.84	37.49	19
III_92	115.96	38.01	18
III_93	116.04	38.03	16
III_94	116.06	38.03	17
III_95	116.11	38.2	15
III_96	116.11	38.22	14
III_97	116.01	39.1	15
III_98	115.98	39.11	19
III_99	115.48	38.7	20

**Fig. 2:**
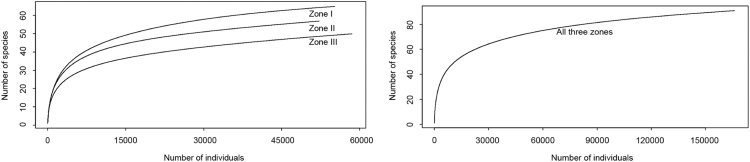
Rarefaction curves for identified rotifer species in different zones. Left - the each zone, right - the combined set of 94 samples [2].

For each rotifer sample, species identification was conducted in three representative subsamples. All individuals of rotifers in each subsample were identified and counted under a microscope (Table S2). Rotifers were identified to the species level based on available taxonomic keys [8], except for several genera such as *Synchacta* and *Trichocerca* (identified to the genus level). In addition, two crucial taxonomic groups on rotifers’ food webs, i.e., protozoans and crustaceans, were counted and considered as biotic environmental variables (Table S1). The adequacy of sampling depth was assessed using species-accumulation curves. These curves clearly showed that the species richness estimated by our methods reached or almost reached to asymptote (Fig. 2).
